# A rapid synthesis of molecularly imprinted polymer nanoparticles for the extraction of performance enhancing drugs (PIEDs)†

**DOI:** 10.1039/d3na00422h

**Published:** 2023-08-28

**Authors:** Mark V. Sullivan, Connor Fletcher, Rachel Armitage, Chester Blackburn, Nicholas W. Turner

**Affiliations:** a Department of Chemistry, Dainton Building, University of Sheffield Brook Hill Sheffield S3 7HF UK mark.sullivan@sheffield.ac.uk n.w.turner@sheffield.ac.uk; b Leicester School of Pharmacy, De Montfort University The Gateway Leicester LE1 9BH UK

## Abstract

It is becoming increasingly more significant to detect and separate hormones from water sources, with the development of synthetic recognition materials becoming an emerging field. The delicate nature of biological recognition materials such as the antibodies means the generation of robust viable synthetic alternatives has become a necessity. Molecularly imprinted nanoparticles (NanoMIPs) are an exciting class that has shown promise due the generation of high-affinity and specific materials. While nanoMIPs offer high affinity, robustness and reusability, their production can be tricky and laborious. Here we have developed a simple and rapid microwaveable suspension polymerisation technique to produce nanoMIPs for two related classes of drug targets, Selective Androgen Receptor Modulators (SARMs) and steroids. These nanoMIPs were produced using one-pot microwave synthesis with methacrylic acid (MAA) as the functional monomer and ethylene glycol dimethacrylate (EGDMA) as a suitable cross-linker, producing particles of an approximate range of 120–140 nm. With the SARMs-based nanoMIPs being able to rebind 94.08 and 94.46% of their target molecules (andarine, and RAD-140, respectively), while the steroidal-based nanoMIPs were able to rebind 96.62 and 96.80% of their target molecules (estradiol and testosterone, respectively). The affinity of nanoMIPs were investigated using Scatchard analysis, with *K*_a_ values of 6.60 × 10^6^, 1.51 × 10^7^, 1.04 × 10^7^ and 1.51 × 10^7^ M^−1^, for the binding of andarine, RAD-140, estradiol and testosterone, respectively. While the non-imprinted control polymer (NIP) shows a decrease in affinity with *K*_a_ values of 3.40 × 10^4^, 1.01 × 10^4^, 1.83 × 10^4^, and 4.00 × 10^4^ M^−1^, respectively. The nanoMIPs also demonstrated good selectivity and specificity of binding the targets from a complex matrix of river water, showing these functional materials offer multiple uses for trace compound analysis and/or sample clean-up.

## Abstract 2

It is becoming increasingly more significant to detect and separate hormones from water sources, with the development of synthetic recognition materials becoming an emerging field.

## Graphical abstract

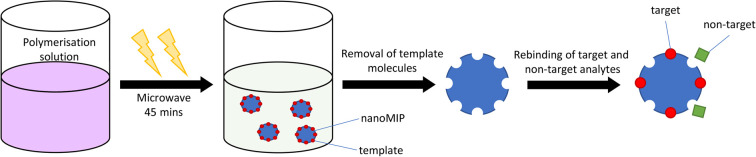

## Introduction

Performance and image enhancing drugs (PIEDs) are a class of substances that are generally abused by not only professional and amateur athletes, but also fitness enthusiasts or students, for body image purposes.^1^ With reported side-effects, that include aggression, depression, liver toxicity and heart issues being reported there is a real concern for a potential global public health issue to emerge.^1–3^ Some of the most frequently abused substances are androgenic anabolic steroids (AASs) and their latest successors, selective androgenic receptor modulators (SARMs). The latter match the desired effects and are easy to come by.^4–7^

Androgenic anabolic steroids (AASs) became widely used as PIEDs, since the first isolation of testosterone and subsequent synthesis of hundreds of synthetic androgens in the 1930's, by elite athletes to vastly improve muscle mass and athletic performance. The performance benefits and associated health risks led them to be placed on the banned substances list by the International Olympic Committee (IOC) in 1976.^8^ Use spread from elite athletes to the general population, and nowadays 4/5's of users do so for image purposes.^9^ It is expected that these users will account for the majority of the future public health problems associated with steroid abuse.^1^

Selective androgen receptor modulators (SARMs) are another class of PIEDs that are currently being misused as performance and image enhancing drugs by athletes and the general public. These unique class of androgen receptor ligands display tissue-selective activation, but exhibit more selectivity in their action.^10,11^ Comparable increases in muscle mass and protein synthesis to AASs are observed but with lesser side-effects.^12^ SARMs are becoming more widely used in both the amateur and competitive elite circuits.^13^ They are recently included in the prohibited substance list by WADA (the World Anti-Doping Agency).^14^

As such monitoring is required, not just within athletes' samples but in the wider environment as the long-term effects of these compounds in not understood. The stable nature of these compounds means they are often found in waste and environmental water samples making water-based epidemiology (WBE) a suitable method for estimating consumption of illicit drug use within the general population, and therefore can be used for monitoring PIED use.^15^

Given the complex nature of these matrices targeted extraction is ideally required to simplify any measurements. General preparative methods exist (*i.e.* solid phase-extraction) to prepare samples for complex chromatographic processes,^16–18^ though these offer limited capabilities for certain family of compounds. Compound-specific tests such as an antibody-based test (*e.g.* ELISA) are suitable for specific detection, but also have limitations,^19–21^ often around cost, stability and batch variations. They also have effectively zero reusability, and test performance is greatly affected by changes in pH, temperature and ionic strength, leading to environmental degradation and denaturation becoming a significant problem.^22,23^ Replacement synthetic recognition materials are therefore an attractive option.

Molecularly imprinted polymers (MIPs) have shown great promise as an alternative to match the performance (selectivity/specificity/affinity) of their biological counterparts while offering performance and robustness in a wide range of conditions. Usually produced using a self-assembly approach, they are simple and cost effective to develop and produce, while offering good integration into modern analytical methods.^24^ The advent of MIP nanoparticles (NanoMIPs) has significantly improved the field by reducing the surface area of the particles and therefore reducing the heterogeneity of potential binding sites.^25–28^ This has allowed for nanoMIPs to be potentially used within biological systems as well as sensor applications. The high surface to volume ratio of these nanoMIPs has allowed for more regular structures to be created which when compared to a traditional bulk MIP provides superior all-round performance, while generating vastly improved yields of effective polymer.^19,20,29,30^

Using a solid-phase synthesis approach is a popular method for producing nanoMIPs and allows for these materials to observe one binding site per nanoparticle, which results the nanoMIPs to offer excellent binding capacities and performances, which are comparable to that of monoclonal antibodies.^31^ The solid-phase approach usually requires a multi-step synthesis, by initially functionalising a solid support, followed by the immobilisation of the target, before the nanoMIP can be produced.^25,32,33^ Even though this method offers high affinity nanoMIPs, it can be time consuming with lower yields than other methods. A suspension polymerisation method is a simple technique whereby polymerisation occurs within a dilute solution, with the MIP nanoparticles precipitating once they have been formed.^34^ This is simple and quick method forms homogenous nanoMIPs and can be tailored to task through changes in solvent, and polymer composition. Furthermore, with a surge of interest in microwave radiation as a thermodynamic driving force, there is the potential for developing environmentally conscious, simple and time efficient methods for synthesis of nanoMIPs.^35,36^

**Fig. 1:**
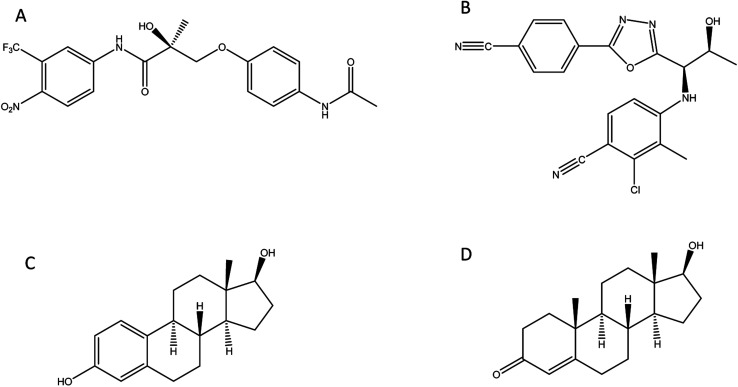
Structure of compounds involved in the study: (A) andarine; (B) RAD-140; (C) estradiol and (D) testosterone.

In this study we investigate the development of MIP nanoparticles (nanoMIPs) using a microwave polymerisation approach for andarine and RAD-140 (Fig. 1A and B) as well as the steroidal targets estradiol and testosterone (Fig. 1C and D), with the two compound families being studied to demonstrate the versatility of this method for imprinting. Whereby, for the first time a new rapid, green, and efficient microwave synthesis is used to produce nanoMIPs. This new technique offers a unique one-pot suspension synthesis, to produce high affinity nanoMIPs recognition materials for SARMs and steroidal targets. This suspension polymerisation technique was chosen in order to prevent unnecessary labour and time loss, while these compounds were chosen as they have been known to be present in river and wastewater.^16,37,38^ Steroids have long been used for imprinting with multiple methods demonstrated including bulk, emulsion, suspension and solid-phase.^39–41^ As such they are an ideal candidate to explore our method. The two selected compounds are bioactive and are found in several pharmaceutical products. With currently only a single MIP study,^29^ SARMs are a novel target for imprinting but as discussed above one that will need addressing in the near future.

## Experimental

### Materials

Acetic acid, azobisisobutytonitrile (AIBN), chloroform, ethylene glycol dimethacrylate (EGDMA), methacrylic acid (MAA), methanol, high molecular weight (146–186 kDa) polyvinyl alcohol (PVA) and toluene were all purchased from Fisher Scientific UK (Loughborough, Leicester, UK). All were of analytical quality or high-performance liquid chromatography (HPLC) grade and used without purification. The templates andarine and RAD-140 were purchased from Biosynth Carbosynth (Compton, Berkshire, UK). Testosterone and estradiol were purchased from Merck (Gillingham, Dorset, UK).

### Instrumentation

A CEM Discover 2.0 microwave synthesizer was used for the production of the imprinted polymers. While a Bruker Alpha FTIR spectrometer was used to obtain the infrared spectra scanning from between 4000–400 cm^−1^, with a resolution of 2 cm^−1^ and 32 scans. The size, shape and surface topography of the MIPS were determined using a Carl Zeiss SEM EVO High Definition 15 Scanning Electron Microscope operating at 10 kV. The samples were mounted on a metal stub with double-sided adhesive tape and gold-coated under vacuum in an argon atmosphere prior to observation. The batch MIP rebinding experiments were performed using UV/vis analysis on a Nanodrop One Spectrophotometer with wavelengths of 230 nm (estradiol), 248 nm (andarine), 275 nm (testosterone), and 300 nm (RAD-140).

### Molecularly imprinted polymer nanoparticle synthesis

To a solution of 0.172 g (2 mmol) of the functional monomer methacrylic acid (MAA) in 10 mL of toluene, 0.25 mmol of a template molecule was added to a 35 mL CEM microwave vial and stirred for 60 minutes, until dissolved. This time allowed for the monomer-template complex to form. Next, 100 mg of PVA was added to 25 mL of double distilled water and stirred at 70 °C until the PVA dissolved, this was then allowed to cool to room temperature and was added to the monomer-template mixture, along with 1.982 g (10 mmol) of EGDMA as a cross-linker and 10 mg (0.06 mmol) of AIBN as the free radical polymerisation initiator. The reaction solution was stirred and degassed with nitrogen for 10 minutes, before being sealed, then placed in a CEM discover 2.0 microwave synthesizer and the reaction was heated up to 110 °C. The reaction mixture was then held at 110 °C for 45 minutes. The resultant polymers were collected, washed initially with acetone twice to remove any unreacted material, on a filter paper. The coagulated nanoparticles were then washed, using Soxhlet extraction for 72 hours with a 9 : 1 solution of methanol:acetic acid, to remove the template. The coagulate was centrifuged in methanol for 5 minutes at 15 000 rpm (RCF: 15 100 × *g*), the supernatant removed, and particles dried. Corresponding non-imprinted polymer (NIP) nanoparticles were produced using the same method, but in the absence of the template. The NIP nanoparticle was used as a control polymer to assess MIP affinity.

### Rebinding studies

The subsequent rebinding effect of the conditioned and equilibrated MIPs and NIPs were characterized using a nanodrop UV/visible spectrometer. The nanoMIPs (20 mg) were placed into Eppendorf tubes containing the target molecule (20 μg), dissolved into 1 mL of double distilled (DD) water. The polymer/target solutions were left for two hours to allow for target rebinding to occur at room temperature (20 ± 2 °C). The mixture was the centrifuged for 5 minutes at 15 000 rpm (RCF: 15 100 × *g*) and the supernatant was then analysed using a NanoDrop One Spectrophotometer at wavelengths 196 nm and 278 nm for andarine and RAD-140, respectively and 250 nm and 290 nm for testosterone and estradiol, respectively. This process was repeated with the corresponding control NIP polymers. The selectivity of the MIPs was studied by investigating the binding of the conditioned nanoMIPs (20 mg) with the corresponding non-templated SARMS and steroid molecules, (20 μg dissolved in 1 mL of DD water). The amount of the target molecule, bound to the polymer B, was calculated by the subtraction of the concentration of the free target molecule, [TM], from the initial target molecule concentration, determined as a mean of three measurements. Scatchard analysis was performed using the binding studies of MIPs with 1 mL of known concentrations (20–100 μg mL^−1^) of the target molecule, with analysis provided by the Scatchard equation (eqn (1)).^42^1
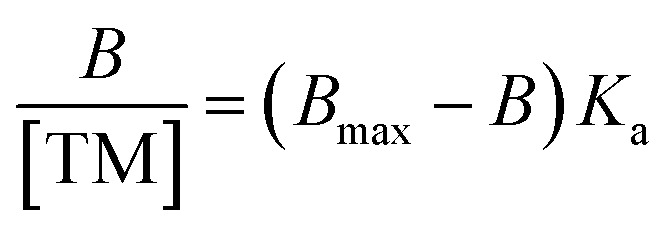
where *K*_a_ is the association constant and *B*_max_ is the theoretical estimate of the maximum number of binding sites. Producing by Scatchard plot (bound concentration/unbound concentration *versus* bound concentration) allows for the determination of the association constant (*K*_a_) *via* the slope of the slope of the line and theoretical maximum number of binding sites (*B*_max_) from the gradient intercept.

Method sensitivity (LOD and LOQ) were calculated by using *σ* (standard deviation of response) and *b* (slope of the calibration curve) and the equations LOD = (3.3 × *σ*)/*b* and LOQ = (10 × *σ*)/*b*.^43^

## Results and discussion

### Molecularly imprinted polymer nanoparticle synthesis

Using a microwave-assisted method with MAA as the functional monomer and EGDMA as the cross-linker, molecularly imprinted polymer nanoparticles (nanoMIPs) were initially synthesised for the molecular recognition of the SARMs targets andarine and RAD-140, as well as the steroidal targets estradiol and testosterone. This new method offers significant advantages over other suspension polymerisation techniques as well as the more commonly solid-phase approach that is currently being used and presented in literature. This is because the time needed for synthesis is rapidly reduced to 45 minutes from 24 hours for the suspension polymerisation and days for the solid-phase technique. Furthermore, the yield is massively increased using the microwave technique, with batches producing nanoMIPs on the gram scale compared with the mg that is produced using the solid-phase technique. This is because a microwave-assisted synthesis allows for accelerated heating of materials because of dielectric heating effects, whereby the microwave energy that is produced is only transferred directly to the reaction components that are susceptible to microwave polarization.^36,44^ By only heating the reaction mixture, energy efficiency is improved and reduces the need to heat any reaction vessels. Due to this direct method of heating the reagents, the time taken for the reaction to reach its activation energy is minimized, reducing the reaction time while also reducing any unwanted side reactions and by-products.^44^

**Fig. 2:**
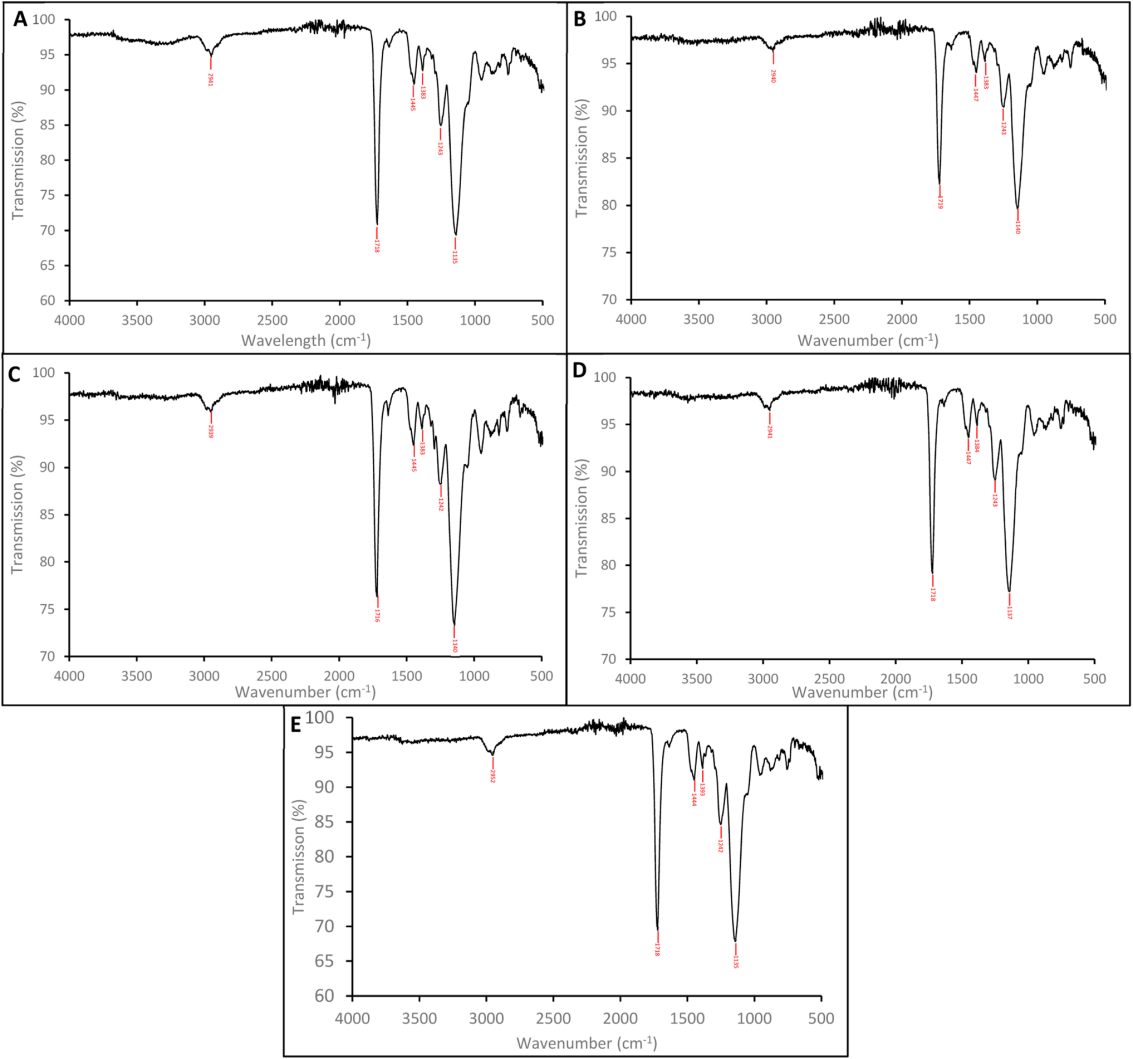
FTIR spectra of the nanoMIPs (and corresponding NIP) for the targets: andarine (A), RAD-140 (B), estradiol (C), testosterone (D) and NIP (E).

The FTIR spectra for the nanoMIPs are shown in Fig. 2A (andarine), 2B (RAD-140) 2C (estradiol) and 2D (testosterone). The FTIR spectrum for the corresponding NIP is shown in Fig. 2E. The O–H stretching at 2941, 2940, 2939, 2941 and 2952 cm^−1^ and the O–H bending vibration 1383, 1383, 1383, 1384 and 1393 cm^−1^ (for Fig. 2A–E, respectively) confirm the presence of carboxylic acid groups (from the methacrylic acid) within the nanoMIP. The occurrence of peaks 1718, 1719, 1716, 1718, and 1718 cm^−1^ (C=O stretch) and 1135, 1140, 1134, 1137 and 1135 cm^−1^ (C–O stretching), for Fig. 2A–E, respectively, show the presence of EGDMA (acting as a crosslinker. The peaks 1445, 1447, 1445, 14 447 and 1444 cm^−1^, (Fig. 2A–E, respectively) show C–H bending vibration of methyl group, mostly likely occurring due the presence of methyl groups in both the methacrylic acid and EGDMA crosslinker. Also shown are the C–O–C asymmetric groups at 1445, 1447, 1445, 14 447 and 1444 cm^−1^ (Fig. 2A–E, respectively), it would be expected to see the C–O–C symmetric groups peaks at approximately 1000 cm^−1^, whereby this is shown as a shoulder (of the strong peak at approximately 1135 cm^−1^), instead of an individual peak. The absence of a C=C double bond stretching (at approximately 1640–1610 cm^−1^), in the spectra confirms the polymerisation of the functional monomer (MAA) and crosslinker (EGDMA). Furthermore, it should be noted that the template/target molecule stretching bands (shown in Fig. S1†), particularly the strong/distinctive bands that would be expected to be shown, are absent from the spectra. This is possible due to the template/target bands being hidden by the polymer bands, especially with the low ratio of template/target compared with the polymer.^24,45^

**Fig. 3:**
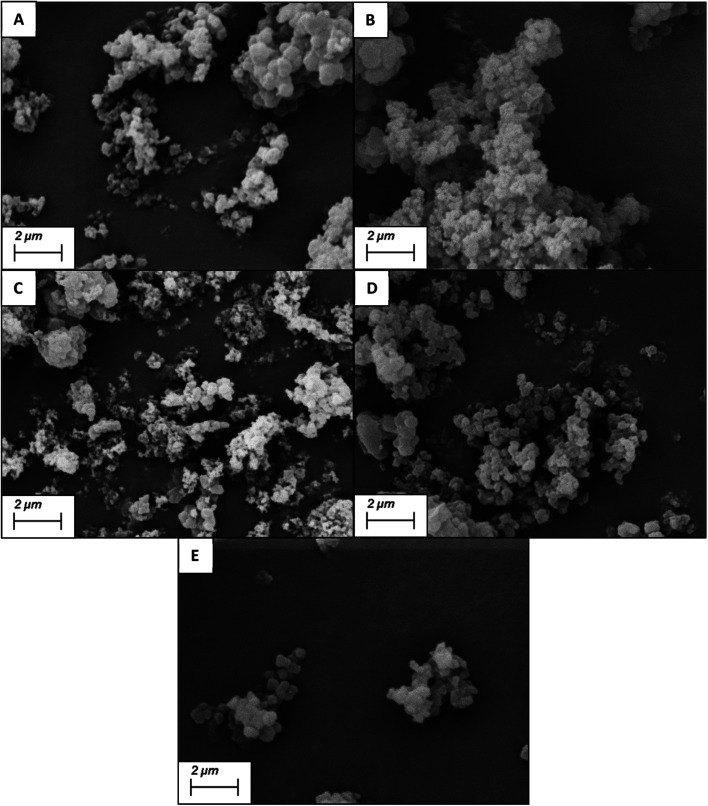
SEM images of the nanoMIPs (and corresponding NIP) for the targets: andarine (A), RAD-140 (B), estradiol (C), testosterone (D) and NIP (E).

The SEM images shows the SARMs and Steroid targeted nanoparticles to be 132.7 (±19.3) nm, 143.3 (±15.4), 120.2 (±18.0) and 135.5 (±13.9) nm for the andarine, RAD-140, estradiol and testosterone nanoMIPs, with the corresponding NIP shows these nanoparticles to 131.5 (±9.1) nm (Fig. 3A–E, respectively). Furthermore, the particles appear to be spherical and dispersive, while forming in clusters. These sizes and patterning are consistent with other protocols for the synthesis of nanoMIPs, particularly the solid-phase method that is commonly used and other suspension polymerisation methods (non-microwaveable and for other target molecules).^27,34,46^

After the subsequent removal of the template from the aggregated (coagulated) nanoparticles *via* Soxhlet extraction, using methanol/acetic acid (9 : 1 v/v), the particles were ready for rebinding studies.

### Molecularly imprinted nanoparticle rebinding studies

**Fig. 4:**
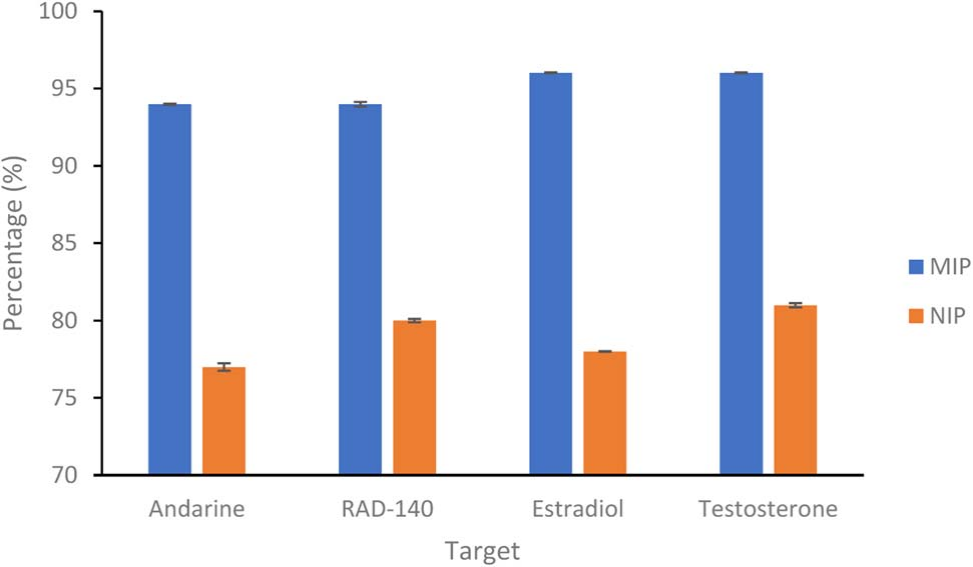
Percentage of SARMs/steroidal targets rebinding to their corresponding nanoMIP or their corresponding NIPs (1 mL of 20 μg mL^−1^ solution with 20 mg of polymer. *N* = 3).

The rebinding of the target molecule is predominately achieved by the same non-covalent interactions (hydrogen bonding, van der Waals and ionic bonding), that were used in the self-assembly of the functional monomers around the template, during the nanoMIP production.^24^ The rebinding performance of microwave synthesised nanoMIPs was measured by using a subtraction technique, whereby a known concentration of the target molecule being mixed with the MIP and being allowed to associate. After centrifugation, the supernatant is then analysed using a Nanodrop One Spectrophotometer, and the amount of target bound was calculated. An initial calibration was plotted by injecting known concentrations (0–70 μg mL^−1^) of the target molecules, then plotting signal response over concentration (Fig. S2†). The percentage rebinding of the targets (andarine, RAD-140, estradiol and testosterone) to the nanoMIPs (and corresponding NIPs) are shown in Fig. 4 and summarised in Table 1. The non-imprinted polymers (NIPs) are themselves cross-linked polymers that are synthesized using the same method (and functional monomers) as the MIP but in the absence of the template. This means they can have the same chemical properties as the MIP but without containing any specific cavities. This means NIPs can exhibit strong non-specific interactions and binding to a range of potential target, whereby these interactions are non-specific. This has resulted in NIP particles being used as a control against MIP particles to compare nonspecific binding to template specific binding. By comparing the nanoMIPs with the corresponding control polymer (NIP) allows for the calculation of an imprinting factor (IF) value and is a commonly used method to determine the strength of interaction of the imprinted polymer towards the target. It is generally accepted that IF values >1.20 deem a MIP to be consider acceptable, while the higher the IF value the greater the selectivity the MIP is towards the target.^47–49^ IF values are calculated using eqn (2), with the calculated IF values also presented (alongside the percentage rebind) in Table 1:2
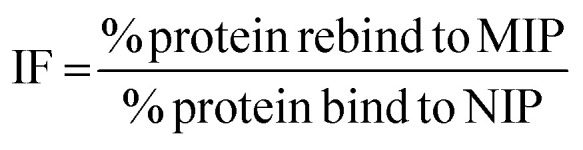


**Table 1:** Percentage of SARMs/steroidal targets rebinding to their corresponding nanoMIP and NIP, and calculated Imprint Factor. (1 mL of 20 μg mL^−1^ solution with 20 mg of polymer. *N* = 3)

NanoMIP	Percentage target bound (%)		IF
	MIP	NIP	
Andarine	94.08 (±0.05)	77.52 (±0.24)	1.22
RAD-140	94.46 (±0.15)	80.37 (±0.11)	1.18
Estradiol	96.62 (±0.03)	78.51 (±0.01)	1.23
Testosterone	96.80 (±0.03)	81.09 (±0.14)	1.19

As shown in Fig. 4 and Table 1, the nanoMIPs offered good affinity for their targets, with a high percentage (all between 94–97%) of the target rebinding to the nanoMIP. The control (NIP) nanoparticles were loaded with the target SARMs molecules to determine if the rebinding is due to a formed MIP cavity and not the polymer. While the NIP shows a relatively high percentage (all between 77–82%) of target molecules binding to the NIP nanoparticles, there is a significant (*p* value of 6 × 10^−6^, *t*-test) decrease in the binding percentage suggesting that this target binding is due to the imprinting effect. The calculated IF values shown in Table 1 and are at the approximate threshold (of 1.2) for an imprinting effect to be considered. While the imprinting factors in Table 1 may seem low we should also factor in the particle density differences. As the control polymers (NIPs) are absent of cavities, there is the potential for these particles are denser than the corresponding MIPs, resulting in more functional monomers contained within the same volume. Given these are effectively spherical materials, an equal mass of NIP could have a greater functionality over the particle surface compared with the MIP, where the main functionality is contained within the recognition cavity.^47^ While the non-specific electrostatic interactions should be the same as it is the same material, we can hypothesise that the actual imprinting effect is larger than this data suggests.

As studies have shown NIPs to have different behaviour to MIPs, caused by a difference in morphology, with the presence of the template during polymerisation affecting the rate of reaction and polymer porosity. The use of a selectivity factor (SF) has now become the more preferential method for assessing the binding ability of the MIP this is calculated using eqn (3), where the binding of the target analyte is compared to a non-target analyte.^47–49^ The selectivity of the nanoMIPs was explored by studying their binding with non-target SARMs and steroid molecules, chosen due to similarity is size, structure and use.3
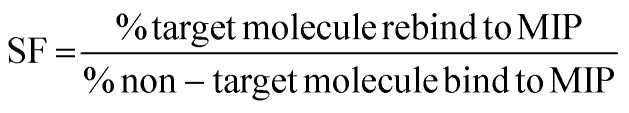


**Fig. 5:**
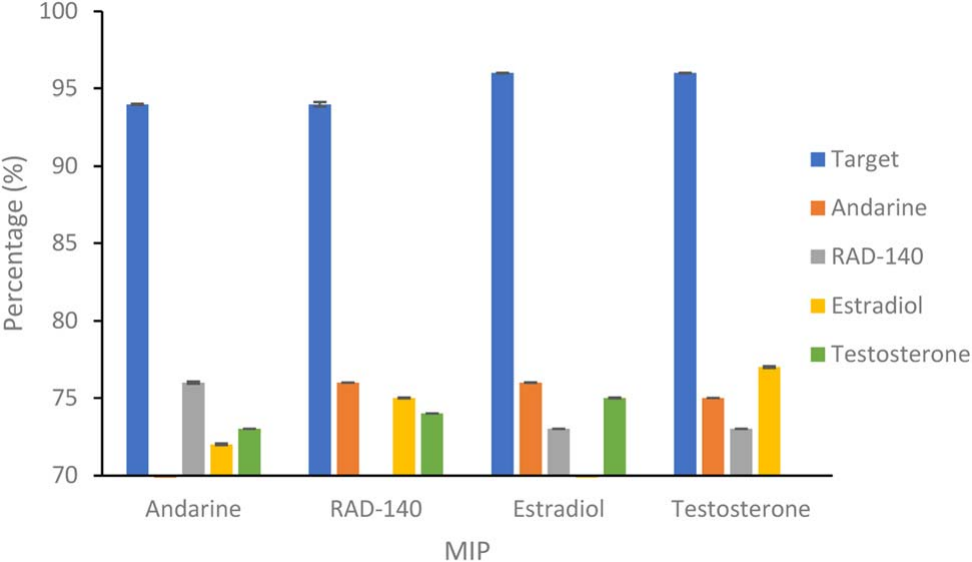
Percentage of SARMs/steroidal targets and non-targets rebinding to 20 mg of respective nanoMIP at 20 μg mL^−1^. *N* = 3.

The binding of the non-target molecules to the nanoMIPs (Fig. 5 and Table 2), produced a slight improvement in the results compared to that of the target molecule binding the NIP control polymer, with binding of the non-target molecules, with a range of 72–78% binding (compared with 77–82% for the binding of target molecules to the NIPs) of the non-target molecules, with a *p* value of 0.01 (Anova test). Using the selectivity factor (SF) values, presented in Table 3, as a more suitable measure of assessing MIP performance, shows improvements, with all SF values above the 1.2 threshold that deems MIPs to be considered acceptable.

**Table 2:** Percentage binding of SARMs/steroidal target to a non-corresponding nanoMIPs (1 mL of 20 μg mL^−1^ solution with 20 mg of polymer. *N* = 3)

MIP	Percentage non-target bound (%)			
	Andarine	RAD-140	Estradiol	Testosterone
Andarine		76.58 (±0.08)	72.80 (±0.06)	73.29 (±0.01)
RAD-140	79.09 (±0.01)		75.68 (±0.03)	74.95 (±0.01)
Estradiol	76.62 (±0.02)	73.21 (±0.01)		75.20 (±0.03)
Testosterone	75.46 (±0.01)	73.27 (±0.01)	77.41 (±0.07)	

**Table 3:** The selectivity factor (SF) values for the non-target SARMs and steroidal molecules binding to the MIPs

MIP	SF Values			
	Andarine	RAD-140	Estradiol	Testosterone
Andarine		1.24	1.31	1.29
RAD-140	1.24		1.25	1.27
Estradiol	1.26	1.32		1.28
Testosterone	1.28	1.32	1.25	

The binding behaviour of the nanoMIPs (and their corresponding NIPs) was investigated using batch rebinding, with association constants (*K*_a_ values) of the polymers estimated with the Scatchard equation (eqn (1)). The Scatchard plots for the MIPs and their corresponding NIPs are presented in Fig. S3 and S4† (nanoMIP and NIP, respectively) and display linear transformations, with the slope of line representing the association constant (*K*_a_). These *K*_a_ values are presented in Table 4.

**Table 4:** Association constant (*K*_a_) values for the binding of the SARMS and Steroid molecules to their corresponding nanoMIP. and NIP

	*K*_a_ values (M^−1^)	
Target	MIP	NIP
Andarine	6.60 × 10^6^	3.40 × 10^4^
RAD-140	1.51 × 10^7^	1.01 × 10^4^
Estradiol	1.04 × 10^7^	1.83 × 10^4^
Testosterone	1.51 × 10^7^	4.00 × 10^4^

As shown in Table 4, the control polymers (NIPs) have *K*_a_ values of 3.40 × 10^4^ M^−1^ (andarine), 1.01 × 10^4^ M^−1^ (RAD-140), 1.83 × 10^4^ M^−1^ (estradiol), and 4.00 × 10^4^ M^−1^ (testosterone), which shows that NIP has minimal affinity towards the target molecules. The formation of specific cavities within the polymer matrix greatly increases the affinity of the polymer, with the nanoMIPs increasing in affinity for their respective targets with approximate increases of 190-fold for andarine (*K*_a_ values from 3.40 × 10^4^ M^−1^ to 6.60 × 10^6^ M^−1^), 1500-fold for RAD-140 (*K*_a_ values from 1.01 × 10^4^ M^−1^ to 1.51 × 10^7^ M^−1^), 570-fold for estradiol (*K*_a_ values from 1.01 × 10^4^ M^−1^ to 1.51 × 10^7^ M^−1^), and 380-fold for testosterone (*K*_a_ values from 1.01 × 10^4^ M^−1^ to 1.51 × 10^7^ M^−1^). The increases in affinity, from NIP to MIP, are to be expected and shows that the cavities created during the self-assembly polymerisation process, have specific recognition for the target and locks the molecule into place.

### Extraction from river water samples

The ability of the nanoMIPs to selectivity bind these target compounds from complex water samples is important as this allows for the understanding of community drug use through WBE and other environmental tracing. We investigate this through repeating the extraction using river water samples, collected from the river soar at co-ordinates 52°37′51.2′′N, 1°08′32.7′′W. The collected water was initially filtered through a 0.22 μm filter to remove sediment and organic matter (bacteria *etc.*) and then spiked with 20 μg mL^−1^ of either the SARMs or steroidal compounds. To 20 mg of the corresponding nanoMIP, 1 mL of the spiked sample, and the amount of the target bound to the nanoMIPs was calculated using the previous extraction method and the calculated using the river water calibration curves presented in Fig. S5.† The percentage of the target analyte bound to the nanoMIPs is summarised in Table 5.

**Table 5:** Percentage of SARMs/steroidal target rebinding to their corresponding nanoMIP from a river water sample (1 mL of 20 μg mL^−1^ solution with 20 mg of polymer. *N* = 3)

NanoMIP	Percentage target bound (%)
Andarine	95.12 (±0.14)
RAD-140	92.27 (±0.31)
Estradiol	93.09 (±0.28)
Testosterone	94.45 (±0.46)

Table 5 shows the nanoMIPs demonstrated the high ability to rebind and collect their imprinted targets from river water samples. This was consistent with the amount of target rebound within the initial model studies and shows that the complex media of the river water samples does not have any interfering effect on the recognition, allowing the nanoMIPs to bind analytes within complex media.

The theoretical LOD and LOQ validation for this methodology calculated according to Choudhari *et al.*^43^ with the LOD found to be 1.42 μg mL^−1^, 3.56 μg mL^−1^, 3.36 μg mL^−1^, and 2.74 μg mL^−1^, for the rebinding of andarine, Rad-140, estradiol, and testosterone, from water, respectively. While the LOD were found to be 1.41 μg mL^−1^, 3.60 μg mL^−1^, 2.99 μg mL^−1^, and 3.43 μg mL^−1^, for the rebinding of andarine, Rad-140, estradiol, and testosterone, from river water, respectively. The LOQ found to be 4.30 μg mL^−1^, 9.77 μg mL^−1^, 9.19 μg mL^−1^, and 8.31 μg mL^−1^, for the rebinding of andarine, Rad-140, estradiol, and testosterone, from water, respectively. While the LOQ were found to be 4.28 μg mL^−1^, 9.93 μg mL^−1^, 9.08 μg mL^−1^, and 9.42 μg mL^−1^, for the rebinding of andarine, Rad-140, estradiol, and testosterone, from river water, respectively.

## Conclusion

Using a new microwave methodology, we have demonstrated for the first time a simple, rapid synthesis of nanoMIPs *via* a suspension polymerisation method for two classes of compounds that require monitoring. A basic methacrylic acid (functional monomer) and EGDMA (cross-linking agent) system was used as a demonstrator. In all cases uniform MIP nanoparticles were produced with comparable sizes ranging from 120–143 nm.

The nanoMIPS produced were shown to exhibit good capacity and selectivity for their target molecules when tested against a control non-imprinted polymer (NIP). The imprinting factors for all polymers were over the recommend 1.2 threshold ratio, thus showing a good MIP effect. The improved selectivity factor was also investigated by binding non-targets to the nanoMIPs, with SF values for the nanoMIPs all being over the recommended 1.2 threshold, thus showing that the nanoMIPS offer specificity. Additionally, the nanoMIPs showed good recognition with association constants (*K*_a_ values) in micromolar range (1.04 × 10^7^–6.60 × 10^6^ M), an approximate 100-fold improvement over the NIP nanoparticle. The nanoMIPs were also able to rebind compounds to the same level from the complex media of river water highlighting potential applications in analytical methodologies as clean-up and capture materials.

This is a simple proof-of-concept study, which demonstrates the ease of production. There are multiple areas whereby this work could be further developed to improve the performance of the polymers and is currently being explored within our follow-on work. We are exploring the use of *in silico* methodology, to optimise polymer composition towards improving MIP selectivity and affinity; and investigating the polymerisation reaction conditions to further study and control the size distribution of the nanoparticles.

Furthermore, the use of different matrices, targets, and analytical instrumentation, to improve sensitivity is also underway. We envisage that these functional nanomaterials, that offer chemical selectivity could play an interested part in the future of analytical methodology, especially within solid phase extraction. Similarly, due to the small size (in the nanometre scale) of these materials opens to the suggestion that they could be used for therapeutics and labelling.

## Author contributions

Mark Sullivan: conceptualization, formal analysis, investigation, methodology, writing – original draft preparation. Connor Fletcher: investigation, methodology. Rachel Armitage: investigation, methodology. Chester Blackburn: formal analysis, writing – original draft preparation. Nicholas Turner: conceptualization, writing – original draft, preparation, supervision, funding acquisition.

## Conflicts of interest

The authors declare no conflicts of interest.

## Supplementary Material

NA-005-D3NA00422H-s001
