# Supplementary material for: A rapid synthesis of molecularly imprinted polymer nanoparticles for the extraction of performance enhancing drugs (PIEDs)
Source: Nanoscale Adv. 2023 Aug 28;5(19):5352–60. doi: 10.1039/d3na00422h (PMC10521259; doi:10.1039/d3na00422h)
Supplement: NA-005-D3NA00422H-s001 [file NA-005-D3NA00422H-s001.pdf]

Electronic Supplementary Material (ESI) for Nanoscale Advances.
This journal is © The Royal Society of Chemistry 2023

A Rapid Synthesis of Molecularly Imprinted Polymer Nanoparticles for the Extraction of Performance Enhancing Drugs (PIEDs).

Mark V. Sullivan[ab*], Connor Fletcher[b], Rachel Armitage[bc], Chester Blackburn[ab], Nicholas W. Turner[ab*]

[a] Department of Chemistry, Dainton Building, University of Sheffield, Brook Hill, Sheffield S3 7HF, United Kingdom.

[b] Leicester School of Pharmacy, De Montfort University, The Gateway, Leicester, LE1 9BH, United Kingdom.

[c] School of Archaeology and Ancient History, University of Leicester, University Road, Leicester, LE1 7RH

*n.w.turner@sheffield.ac.uk

*mark.sullivan@sheffield.ac.uk

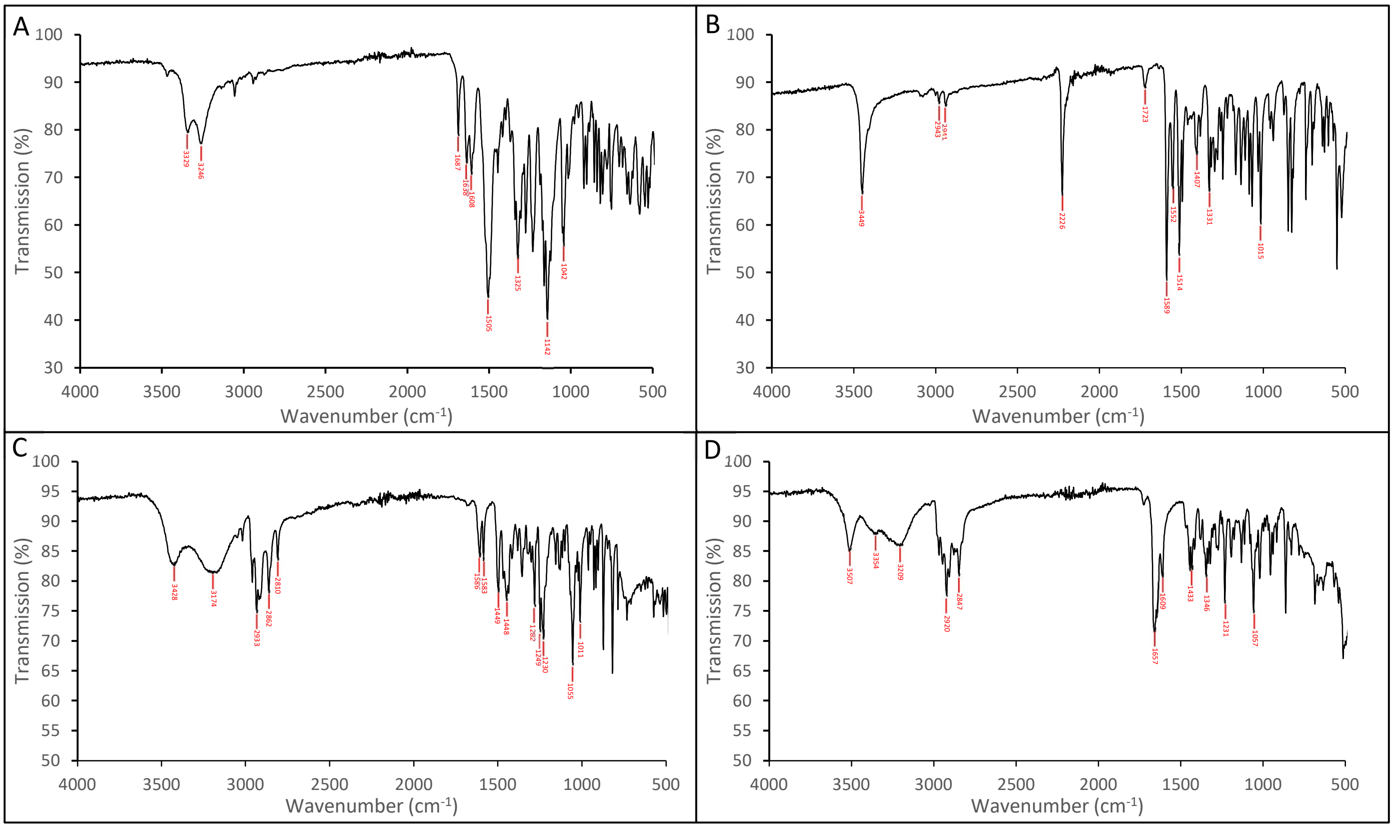

Figure S1: FTIR spectra of the imprinted target molecules: Andarine (A), RAD-140 (B), Estradiol (C) and Gestrinone (D), obtained using a Bruker FT-IR spectrometer (alpha model).

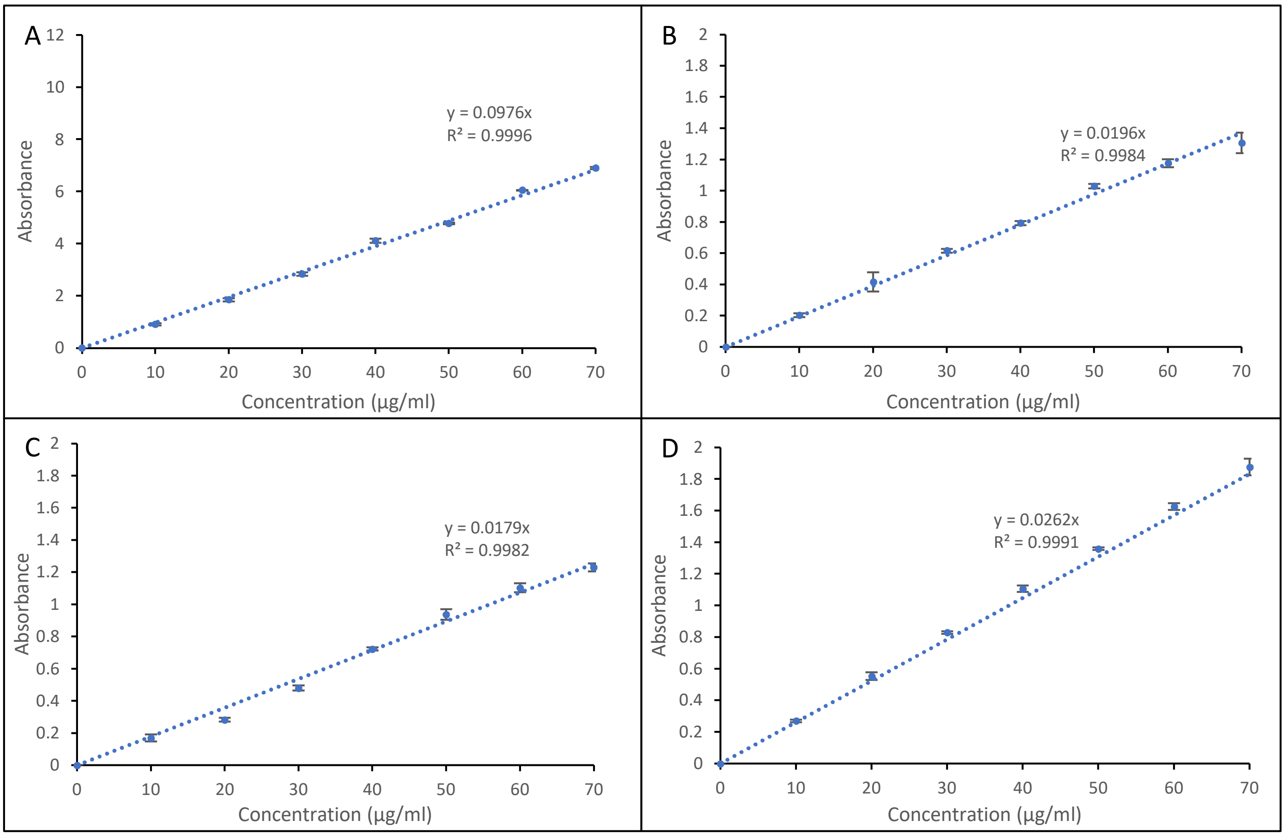

Figure S2: Calibration plots of the target molecules: Andarine (A), RAD-140 (B), Estradiol (C) and Testosterone (D), obtained using a Nanodrop One Spectrophotometer.

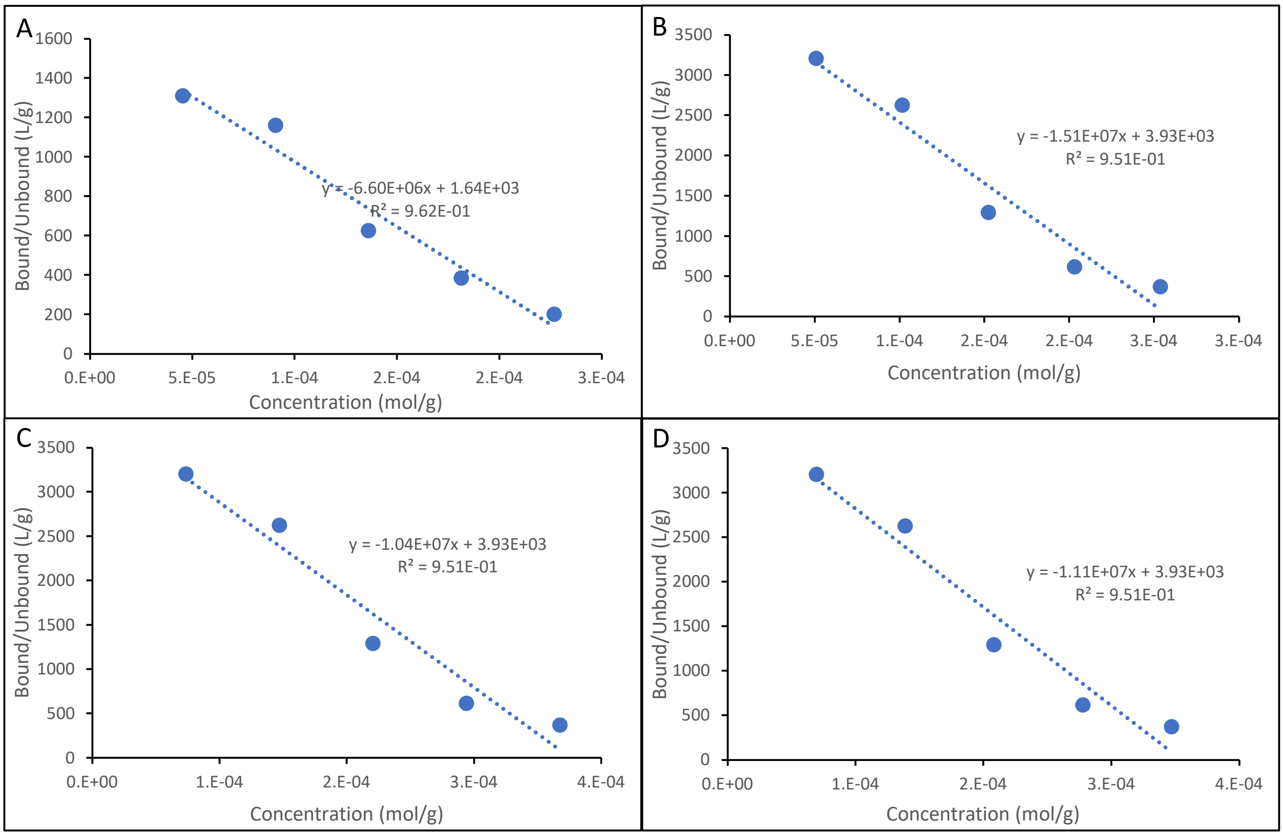

Figure S3: Scatchard analysis plot for the rebinding of the target molecules to their corresponding nanoMIPS: andarine (A), RAD-140 (B), estradiol (C), testosterone (D).

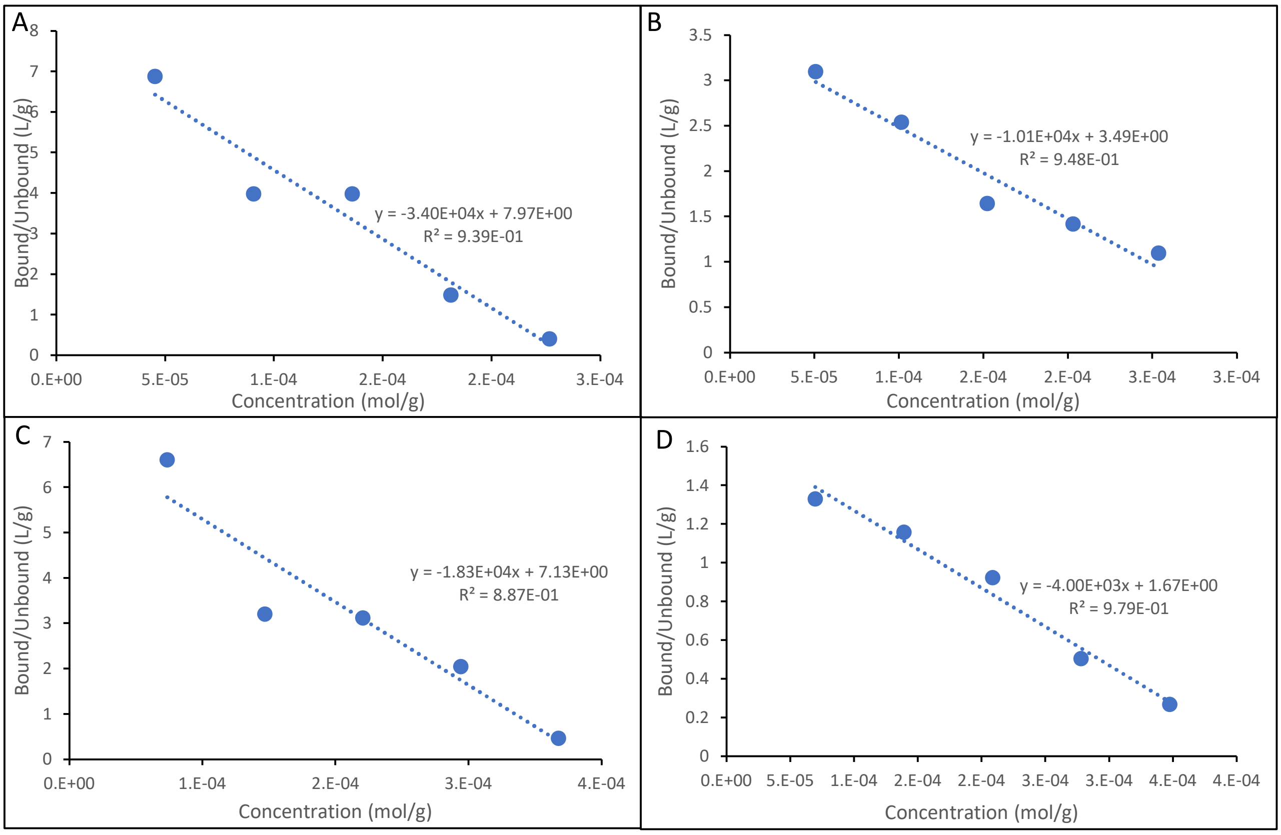

Figure S4: Scatchard analysis plot for the binding of the target molecules to the control non-imprinted polymers (NIPs): andarine (A), RAD-140 (B), estradiol (C), testosterone (D).

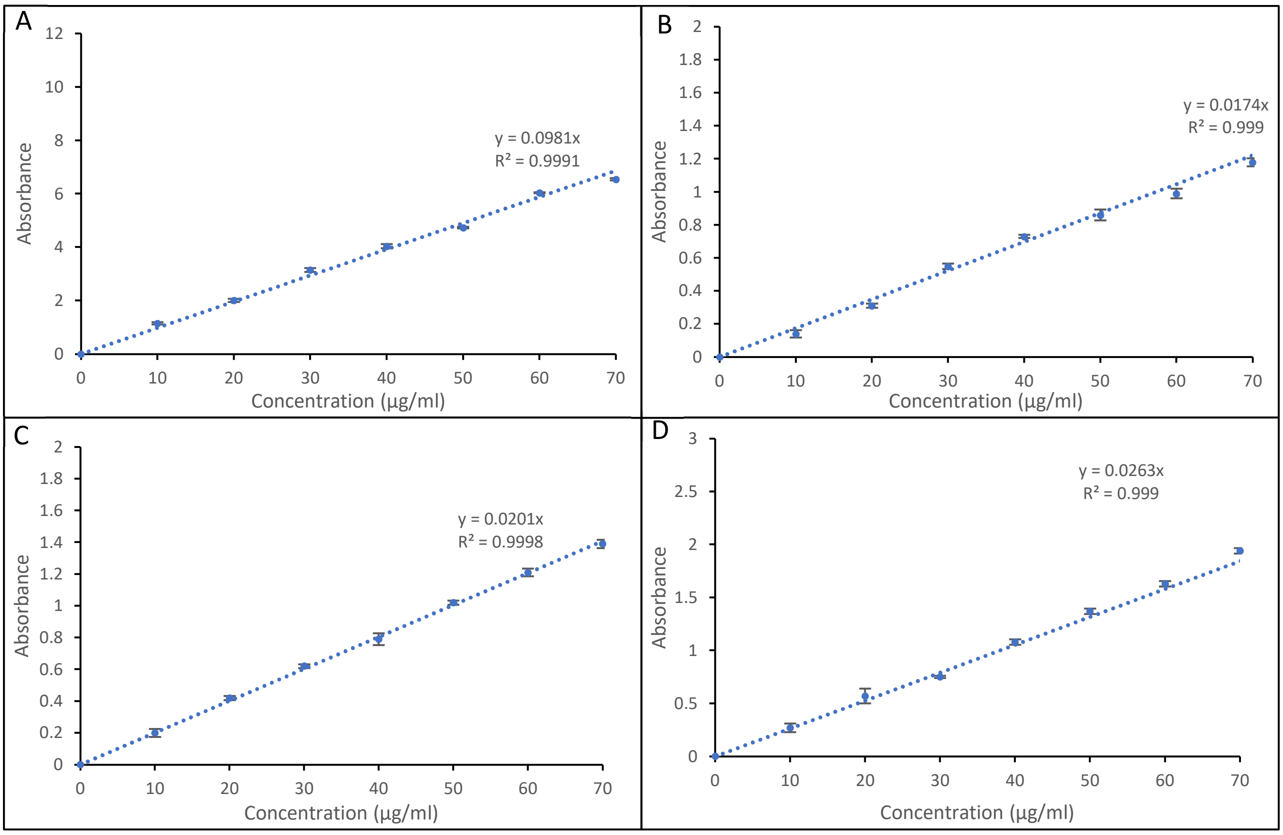

Figure S5: Calibration plots of the target molecules: Andarine (A), RAD-140 (B), Estradiol (C) and Testosterone (D), in River Water, obtained using a Nanodrop One Spectrophotometer.
